# Transitions of the Bacteria–Fungi Microbiomes Associated with Different Life Cycle Stages of Dinoflagellate *Scrippsiella acuminata*

**DOI:** 10.3390/microorganisms13061340

**Published:** 2025-06-09

**Authors:** Caixia Yue, Zhaoyang Chai, Fengting Li, Lixia Shang, Zhangxi Hu, Yunyan Deng, Ying-Zhong Tang

**Affiliations:** 1 CAS Key Laboratory of Marine Ecology and Environmental Sciences, Institute of Oceanology, Chinese Academy of Sciences, Qingdao 266071, China; zhaoyangchai@qdio.ac.cn (Z.C.); lifengting@qdio.ac.cn (F.L.); lxshang@qdio.ac.cn (L.S.); huzx@gdou.edu.cn (Z.H.); 2 Animal, Plant and Food Inspection Center of Nanjing Customs District, Nanjing 210019, China; 3 Laboratory for Marine Ecology and Environmental Science, Qingdao Marine Science and Technology Center, Qingdao 266071, China; 4 Center for Ocean Mega-Science, Chinese Academy of Sciences, Qingdao 266071, China

**Keywords:** dinoflagellate, resting cysts, host-attached community, free-living community, core bacterial microbiome, phosphate solubilizing microbiome

## Abstract

Dinoflagellates significantly contribute to the carbon fixation and microbial loop in the ocean with high ecological diversity. While the microbial communities associated with the HABs of dinoflagellates have attracted intensive attention in recent years, little attention has been paid to the microbiomes associated with resting cysts, an important stage in the life cycle and bloom initiation dynamics of dinoflagellates. Using *Scrippsiella acuminata* as a representative of cyst producers and cyst-relevant research in dinoflagellates, we surveyed the bacteria and fungi microbiomes long associated with different life cycle stages of the dinoflagellate culture through 16S and ITS rRNA amplicon sequencing, and predicted their possible functions using the PICRUSt2 algorithm. The results found high species diversity of the associated bacteria–fungi microbiomes, and species featured with diverse and flexible metabolic capabilities that have stably co-occurred with the laboratory culture of *S. acuminata*. The host-attached and the free-living groups of bacteria–fungi microbiomes, as operationally defined in the context, showed significant differences in terms of their nutritional preferences. The bacteria–fungi species diversity and community structure associated with cysts are also distinguished significantly from that with vegetative cells, with the latter attracting more bacteria–fungi species specializing in phosphate solubilization. These results suggest that the relative species abundance and thus the community structure of the host-associated microbiome shift with the transition of life cycle stages and environmental conditions. Our findings show the association tightness between bacteria–fungi microbiomes and dinoflagellate hosts and the different life stages of hosts shaping the bacteria–fungi communities, which result in dynamic and specific interactions between bacteria–fungi microbiomes and their hosts.

## 1. Introduction

In aquatic environments, phytoplankton are closely associated with heterotrophic microbes including viruses, bacteria, and fungi, which play key roles in the physiology and ecology of phytoplankton [1]. These microorganisms (referring to bacteria and fungi in the context of this work) in the phycosphere are crucial in phytoplankton growth, population succession, and biomass decay, even strongly influence the carbon, nitrogen, phosphorus, and sulfur cycling in aquatic habitats, regulate marine ecosystem functions, and promote global biogeochemical cycles [2,3,4,5]. Among all the microbes in the phycosphere, bacteria have historically received more extensive attention, which arguably represent the most important inter-organism association in aquatic environments [2]. However, in spite of that, fungi (marine species in particular) have been traditionally less studied for their roles in the phycosphere, most recent studies have indicated that, as compared to bacteria, fungi may play equally important roles in organic matter cycling and food web dynamics [6], and thus, there has been increasing interest in exploring fungi in marine environments. On the other hand, our knowledge about the species diversity and understanding of the ecological functions of marine fungi remain extremely limited [6,7]. A number of studies have shown that the interactions between phytoplankton and their associated bacteria have been considered to be a unique mechanism for macronutrients and micronutrients’ acquisition and growth regulation [1,2,7,8,9,10,11,12]. Another study has found that the phytoplankton biomass and production necessarily depend on the active metabolism of heterotrophic bacterial communities even when inorganic nutrients are adequate, suggesting that the presence of bacteria is critical for phytoplankton growth and production [13]. Geng and Belas [14] also reported that the axenic culture of the heterotrophic dinoflagellate *Pfiesteria piscicida* could not grow and eventually died without bacteria, but the culture could restore growth with the addition of a mixed bacterial assemblage. Fortin et al. [15] found that the blooms of the dinoflagellates *Margalefidinium polykrikoides* and *Alexandrium monilatum* impact the estuarine microbiome in different ways. The associations between phytoplankton and their microbiomes thus could strongly influence the physiology of both sides, and the mechanisms of these mutualistic interactions and their ecological functions deserve more extensive and intensive investigations.

Phytoplankton, including about a half of all dinoflagellates, possess many common features that allow them to flourish in the water column, including flagella-aided vertical and horizontal migrations, floating according to varying buoyancy, mixotrophy (thus called “mixoplankton”) [16], toxicity to predators, and allelopathy to competitors [17,18,19,20,21,22]. As the second most abundant eukaryotic phytoplankton, dinoflagellates significantly contribute to the carbon fixation and microbial loop in the ocean with high ecological diversity [23,24]. More importantly, dinoflagellates are responsible for more than 75% of harmful algal bloom (HAB) events [25] and constitute about 54% of all known toxic microalgae [26]. Therefore, dinoflagellates have been intensively examined from multiple facets for their biology and ecology. While the growth of dinoflagellates or HAB dynamics are primarily controlled by physical factors and inorganic nutrients, a long-neglected but also important aspect is the biotic factors such as the interactions between HAB-causing species and their associated microbiomes [27]. Within the phycosphere, microbiomes can benefit the dinoflagellate cells through regenerating inorganic nutrients, increasing the iron solubility, and providing essential compounds such as vitamins B_1_ and B_12_ [10,11,28,29,30]. Furthermore, previous studies found that certain dominant bacterial clusters (such as *Roseobacter*, *Flavobacterium*, *Marinobacter*, and *Alteromonas*) were commonly associated with the phycosphere of dinoflagellates in both the field samples [31,32,33,34] and laboratory cultures [35,36,37,38,39,40,41,42]. These apparently common patterns indicate that the specific relationship would have been established between dinoflagellates and bacteria with particular lifestyles and functions. However, the relationships between phytoplankton and the surrounding microbiomes may be more complex and dynamic than what have been generalized. Many studies found that the dynamic patterns of the microbial community were closely related to the bloom cycle of microalgae, indicating the existence of physical associations and physiological interactions between bacteria/fungi and microalgae [31,32,33,34,43,44,45,46,47,48,49,50,51,52]. Some other case studies have shown that the community composition of the associated bacteria dynamically changed with the growth stage of the host phytoplankton [37,38,40,53,54]. More recently, several laboratory studies have even focused on the differences between the attached and free-living bacterial communities [38,55,56], because the attached and free-living communities (although not well defined in the literature up to date) were considered to have different interaction mechanisms with the host, in which the attached communities always have a closer association with the host [57]. Nevertheless, more studies mixed them together, using the term “associated” to refer to all bacteria and fungi related to algal cells rather than attempting to distinguish between the attached and free-living communities. Therefore, the global overview of the abundance and composition of the attached and free-living microbial communities in different life stages of a particular species is helpful to comprehensively understand the interactions between this species and their related microbiomes.

Many phytoplankton species have a resting stage in their life histories, as observed in dinoflagellates, diatoms, chlorophytes, cyanobacteria, chrysophytes, haptophytes, cryptophytes, raphidophytes, and euglenophytes. In dinoflagellates, the resting stage cells are called resting cysts, which are usually formed via sexual processes, function like the seeds of higher plants, exist as a dormant stage in the sediment, and importantly, play a vital role in the biology and ecology of dinoflagellates, especially for the HAB-causing species [22,58,59]. Given the mounting investigations on the associated microbiomes of phytoplankton and HAB species as briefed above and the vital importance of resting cysts, no study, to our knowledge, has ever particularly focused on the microbiomes associated with resting cysts, except for some that are loosely relevant to resting cysts. Examples include isolations of bacteria with cyst formation-promoting or cyst formation-inhibiting activity from the toxic dinoflagellates *Alexandrium* and *Lingulolaux polyedra* (=*Lingulodinium polyedrum*) [60,61,62,63,64]. These works aimed to examine the effects of bacteria as a “biotic factor” on the cyst formation. After all, up to date, little has been known about the compositions and possible functions of the bacteria–fungi microbiomes associated with the dormancy maintenance of resting cysts, a keystone stage in the life history (life cycle) of dinoflagellates.

The dinoflagellate *Scrippsiella acuminata* (Ehrenberg) Kretschmann, Elbrächter, Zinssmeister, S. Soehner, Kirsch, Kusber & Gottschling [=*Scrippsiella trochoidea* in Loeblich, 1976; see [65]] is a cosmopolitan HAB-causing species, with blooms [66] and cysts [67,68] observed globally. The *S. acuminata* exhibits a typical dinoflagellate life cycle, including a vegetative cell stage (n) and a sexually produced resting cyst stage (2n). While vegetative cells (n) reproduce via longitudinal binary division in water, they may form haploid gametes, and the fusion of two gametes form motile, diploid planozygotes, which either undergo meiotic division and return to the vegetative stage (n), or eventually form resting cyst (2n) and sink to the surface of sediment. In general, resting cysts of *S. acuminata* have calcareous spines on their surface and red accumulation bodies within the cysts [69]. This species produces resting cysts more easily than most of the other dinoflagellates both in the field and laboratory, and thus has been adopted as a representative in life history- and cyst-relevant studies [70,71,72,73,74]. Most recently, we preliminarily observed that the resting cysts of the dinoflagellate *S. acuminata* hosted bacterial assemblages that are of diverse trophic strategies under conditions typically observed in marine sediments such as low temperature, darkness, and anoxia [75]. In this study, we investigated the diversity and composition of the host-attached and free-living bacteria–fungi microbiomes associated with the laboratory-cultured *S. acuminata* cells at different stages of their life cycle (i.e., vegetative cells and cysts) under different physiological conditions (e.g., different time periods of storage, temperatures, and oxygen levels), through high-throughput gene amplicon sequencing for the 16S rRNA gene (for bacteria) and ITS region (for fungi). We believe that the present work provides a novel perspective of the bacterial and fungal communities associated with the whole life cycle of *S. acuminata*, and the unique insights into the phycosphere microbiomes associated with the dormancy maintenance of resting cysts and their potential ecological implications. Notably, our work demonstrates the possible active interactions between the dormant resting cysts and the associated bacteria–fungi microbiomes and thus opens a window for further investigations on the functions of microbiomes during the dormancy of cysts in the field.

## 2. Materials and Methods

### 2.1. Establishment of an Algal Culture and Maintenance, and Resting Cysts’ Production

The clonal culture of *S. acuminata* (strain number IOCAS-St-1) used in this study was isolated from the Yellow Sea and provided by the Marine Biological Culture Collection Center, Institute of Oceanology, Chinese Academy of Sciences (IOCAS). The culture has been cultured in the laboratory for 13 years since its establishment and maintained in sterile natural seawater-made f/2-Si medium [76] with a salinity of 32–33 under a temperature of 15 or 21 °C, an irradiance of ~100 µmol photons m^−2^ s^−1^, and a photoperiod of 12:12 h (light/dark) in an incubator. These culturing conditions were also used in the following experiments unless otherwise stated. Vegetative cells at an exponential growth stage were maintained in 75 cm^2^ cell culture flasks (Sorfa, Huzhou, China) with only 1/1000 of the nitrogen and phosphorus concentrations of normal artificial seawater-made f/2 medium. Resting cysts were produced and harvested from the abovementioned cultures that had been incubated for 15 days, and washed several times with sterile artificial seawater in six-well culture plates to remove all vegetative cells as checked under an inverted microscope (IX73, Olympus, Tokyo, Japan) [77]. The resting cysts used in this study had typical calcareous spines and contained a red accumulation body. Note that all bacterial and fungal species detected to be associated with the dinoflagellate as described below are assumed to come along with the initial single cell of *S. acuminata* from which the culture was established, since all procedures of the culture maintenance had been conducted under axenic conditions in the laminar flow hood and all the batches of culture media were sterilized. Consequently, all the changes in bacterial and fungal community structure observed in different types of samples were also considered to be the changes in the relative abundance of species within the initial microbiome associated with the clonal culture.

### 2.2. Sample Types and Their Preparations

To obtain host-attached and free-living bacteria and fungi during the whole life history and that at different temperatures, the vegetative cells and cysts of *S. acuminata* were cultured under different temperatures and periods of incubation time (Table 1), with the aims of examining the possible effects of life cycle stage (vegetative growth vs. resting cysts at different conditions) and temperature on the microbiome structure (i.e., the relative abundance of all species) associated with the dinoflagellate. For the vegetative cell samples, vegetative cells in the exponential phase were inoculated into a 75 cm^2^ cell culture flask containing 100 mL of f/2-Si medium with an initial density of ~1 × 10^4^ cells mL^−1^ and then maintained in incubators at 15 °C and 21 °C on a 12:12 h light/dark cycle for 7 days and 20 days prior to sampling. For the resting cyst samples, resting cysts were transferred into a 75 cm^2^ (bottom surface area) culture flask containing 100 mL of f/2-Si medium with a density of ~2 × 10^4^ cells mL^−1^ and stored either in a refrigerator at 4 °C without light, or in incubators set at 15 °C and 21 °C with a normal 12:12 h light/dark cycle for 30 days and 45 days prior to sampling for the separation of different categories of microbiomes and their respective DNA extractions.

To address the potential concerns about experimental design, we particularly point out that, while all other culture parameters (e.g., nutrient composition, salinity, and oxygen) remained rigorously standardized across the treatments, the gradient design incorporating different life cycle stages, incubation time periods, and temperatures provides robust detection of microbiome composition shifts (i.e., non-numerical parameters). Supportive of our point above, it has been demonstrated both theoretically and experimentally that gradient designs could well outperform replicated designs for detecting nonlinear responses [78].

Collection of the “host-attached” bacteria and fungi: All samples of vegetative cells and cysts were firstly centrifuged at 2500× *g* for 2 min (Step 1). According to the method of Su et al. [79], the collected cells and cysts were washed three times and then suspended in 50 mL of sterile natural seawater containing 0.05% Tween-80 and 0.1 M EDTA (for 30 min), with a subsequent addition of 0.5 mg of mL^−1^ lysozyme (for 10 min) and 0.25% SDS (for 10 min) to remove loosely attached microbiomes on the surface. The samples were washed three times with sterilized natural seawater to remove the remaining reagent and then were collected via centrifugation (6800× *g* for 5 min) into 1.5 mL centrifuge tubes and stored at −80 °C until DNA extraction. These samples were categorized as the “host-attached” group (or simply referred to as “attached”) in terms of their microbiomes, which included both the intracellular microbiomes and those tightly attached to the surface of algal cells.

Collection of the “free-living” bacteria and fungi: The supernatants obtained in Step 1 were filtered through a 5 μm pore size fiber membrane (diameter, 47 mm; Millipore, Boston, MA, USA) to remove the algal cells, and the filtrates were then filtered through a 0.2 μm pore size fiber membrane (diameter, 47mm; Millipore) to collect the microbiomes on the membrane, which were categorized as the “free-living” group. The filter membranes were then stored at −80 °C until DNA extraction. Although we centrifuged the samples (2500× *g* for 2 min) in Step 1, most of the free-living bacteria and fungi were still in the culture medium, because the centrifuge speed for collecting the suspended microbiome was usually 12,000× *g* for 5 min [55], a speed about four times higher and a time duration much longer than that applied in Step 1. We categorized the bacteria–fungi assemblages into two groups, namely the host-attached and the free-living groups, with the former including surface-attached and possibly intracellular (i.e., endosymbionts) bacteria–fungi assemblages, and the latter containing free-living or loosely-bound (hence easily detachable) bacteria–fungi assemblages.

### 2.3. DNA Extraction and PCR Amplification of 16S and ITS rRNA Genes

The total DNA of each sample was extracted using the Fast DNA spin kit for soil (mpBio, Thomas Irvine, CA, USA) according to the manufacturer’s protocols, and the quantity and quality of the total DNA were analyzed via 1% agarose gel electrophoresis and using the NanoDrop™2000 spectrophotometer (Thermo Fisher Scientific, Waltham, MA, USA). The V5-V7 hypervariable region of bacterial 16S rDNA was amplified with the primers 799F (5′-AACMGGATTAGATACCCKG-3′) and 1193R (5′-ACGTCATCCCCACCTTCC-3′) [80]. The PCR was carried out on a Mastercycler Gradient (Eppendorf, Hamburg, Germany) using 25 μL of reaction volumes, containing 12.5 μL of 2× Taq PCR MasterMix, 3 μL of BSA (2 ng/μL), 1 μL of Forward Primer (5 μM), 1 μL of Reverse Primer (5 μM), 2 μL of template DNA, and 5.5 μL of ddH2O under the following conditions: 95 °C for 5 min; 28 cycles at 95 °C for 45 s, at 55 °C for 50 s, and at 72 °C for 45 s with a final extension at 72 °C for 10 min [80]. The PCR products were purified using an Agencourt AMPure XP Kit. Deep sequencing (50,000 reads) was performed on the Miseq platform at Allwegene Company (Beijing, China).

The ITS2 region of fungal rDNA (~353 bp fragment) was amplified using the primers fITS7 (5′-GTGARTCATCGAATCTTTG-3′) and ITS4 (5′-TCCTCCGCTTATTGATATGC-3′) [81]. The PCR reactions were conducted in 25 μL mixtures containing 12.5 μL of 2× Phusion^®^ Hot Start Flex Master Mix, 2.5 μL of each primer (1 μM), and 50 ng of template DNA. The cycling parameters were 98 °C for 30 s, followed by 35 cycles of 98 °C for 10 s, 54 °C for 30 s, and 72 °C for 45 s with a final extension at 72 °C for 10 min [82]. The PCR products were collected and purified using the AxyPrep DNA Gel Extraction Kit (Axygen Biosciences, Union City, CA, USA). The purified amplicon libraries were assessed on an Agilent 2100 Bioanalyzer (Agilent, Santa Clara, CA, USA). The libraries were sequenced on the NovaSeq PE250 platform at LC-Bio Technology Company (Hangzhou, China).

### 2.4. Sequence Processing and Bioinformatic Analyses

To obtain high-quality clean data, all the sequences that were less than 200 bp, or had a quality score ≤ 20, or contained ambiguous base calls, were removed. The quality control of raw data was performed in Fqtrim software (v0.9.7) and the chimeric reads were excluded using Vsearch software (version 2.7.1) [83]. The pair-end reads were separated using the sample-specific barcode sequences. The amplicon sequence variants (ASVs, sequences clustered at a similarity level of 100%) were generated with the DADA2 package [84]. The QIIME2 [85] was used to classify bacterial and fungal features into different taxonomic groups (domain, phylum, class, order, family, and genus levels) against the SILVA138 database and UNITE dynamic database [86,87,88]. The richness and diversity indices were analyzed via QIIME2 based on the ASV information [85]. Based on the results of taxonomic annotation and relative abundance, R software (v3.6.0) was used for bar-plot diagram analysis. The β diversity of PCoA (principal coordinate analysis) based on the unweighted unifrac distances were conducted using the QIIME2 process, and the pictures were drawn by using R (v3.6.0). According to the relative abundance table of samples, the Kruskal–Wallis test was used to test the significance of difference at the domain, phylum, class, order, family, and genus levels, so as to identify the important bacteria and fungi in different groups.

### 2.5. Identification of the Core Bacterial and Fungal Taxa

According to previous studies on the core microbiome of dinoflagellates, the core taxa were defined as those taxa highly common (i.e., present in 100% of all samples) and abundant (i.e., the minimum of relative abundance in all samples is ≥0.1%) in different samples [35,55,89,90,91,92].

### 2.6. Prediction of the Functional Potential of the Bacterial and Fungal Microbiomes

The functional inference of bacterial microbiomes was predicted with reference to the KEGG database using the PICRUSt2 (Phylogenetic Investigation of Communities by Reconstruction of Unobserved States) algorithm [93]. The functional annotation of fungal microbiomes was predicted by using PICRUSt2, with references made to the MetaCyc metabolic pathway database [94]. Based on the results of PICRUSt2, the differences between different groups were analyzed by using the STAMP tool [95] subject to a *t*-test. The significance level was set at *p <* 0.05 in all the cases unless otherwise indicated.

## 3. Results

### 3.1. General Descriptions of Bacterial and Fungal Diversity Associated with Scrippsiella acuminata

**Figure 1:**
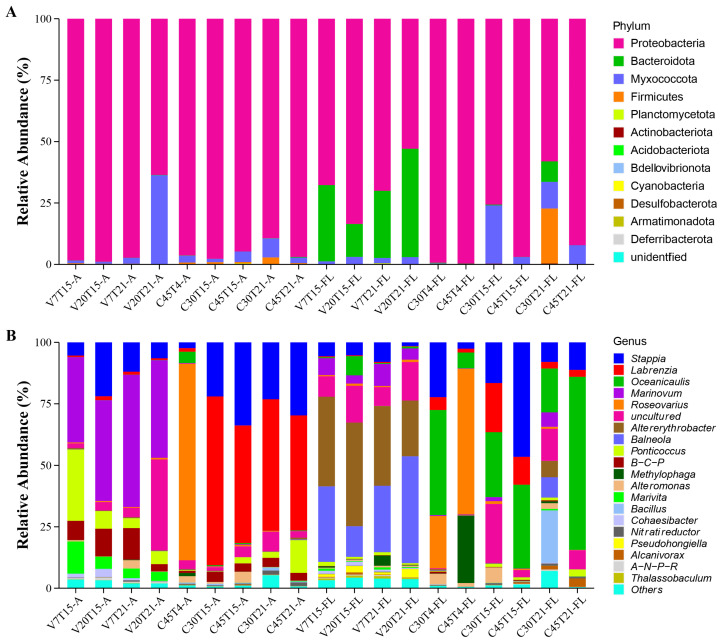
Bacterial community relative abundance of different phyla (**A**) and genera ((**B**), top 20) in the 20 samples. The abundance is presented in terms of percentage in total effective sequences in a sample. The treatment conditions of each sample can be viewed in Table 1. Note: B-C-P, *Burkholderia-Caballeronia-Paraburkholderia*; A-N-P-R, *Allorhizobium-Neorhizobium-Pararhizobium-Rhizobium*; for the sample name below the x-axis V/CXXTXX-A/FL: V, vegetative cell sample; C, cysts sample; XX, the first number is the incubation days; XX, the second number is the incubation temperature; A, host-attached sample; FL: free-living sample.

For bacteria diversity, the 16S rRNA amplicon sequencing obtained 1,501,828 raw reads from 19 samples, with the sample “IOCAS_15” not amplified successfully (Table 1). The raw data was available from the NCBI SRA (Short Read Archive) database (BioProject ID: PRJNA1173930). After filtering short and/or low-quality sequences and removing chimeras, we obtained 1,173,514 clean reads, with effective sequences per sample ranging from 33,017 to 85,074 (Table S1). After the dereplication using DADA2, 1420 Amplicon sequencing variants (ASVs) were obtained. The index Good‘s coverage for each of all the samples was ≥0.99 (Table S2). The effective 1418 ASVs (ASVs annotated as “unclassified” kingdom were filtered out) were annotated to different taxonomic groups from phyla to species via blasting in the SILVA database using the Ribosomal Database Project (RDP) Classifier tool, a naive Bayesian classifier tool. ASVs with more than 97% identity and 100% coverage (unless the reference sequence was shorter than our ASVs) to their respective reference sequences of species were considered as fully identified genera. The bacterial ASVs were assigned to 11 phyla, 19 classes, 67 orders, 109 families, 196 genera, and 223 species (Table S3). The most abundant bacterial phylum was Proteobacteria (85.78%), followed by Bacteroidota (6.62%), Myxococcota (5.96%), and Firmicutes (1.52%, Figure 1A). All the ASVs covered 196 genera (Figure 1B), with *Stappia* (14.99%) and *Labrenzia* (14.05%) being the most abundant, which were followed by *Oceanicaulis* (11.20%) and *Marinovum* (10.63%).

For the fungal species diversity, we obtained 1,594,777 raw reads of ITS sequences from 17 samples, while the three samples “IOCAS_17”, “IOCAS_H”, and “IOCAS_T” were not successfully amplified (Table 1). The raw data was deposited into the NCBI SRA database with the accession number PRJNA1173954. We obtained 1,548,233 clean reads for all 17 samples after removing short and/or low-quality sequences and chimeric sequences (Table S1). The index goods coverage for all the samples was 1.00 except for one sample being 0.99 (Table S2). After dereplication using the DADA2 package, we obtained 521 fungal ASVs, including 335 being host-attached and 261 being free-living (the two groups shared 75 ASVs). The 521 ASVs were annotated to six phyla, 27 classes, 59 orders, 128 families, 203 genera, and 274 species (Table S3). The most abundant phylum was Ascomycota (65.64%), followed by Basidiomycota (33.21%, Figure S1A). At the genus level, *Malassezia* dominated the whole fungal community (21.45%), followed by *Paecilomyces* (7.24%) (Figure S1B).

### 3.2. Common Species Shared by the Attached and Free-Living Groups and Core Genera Determination

**Figure 2:**
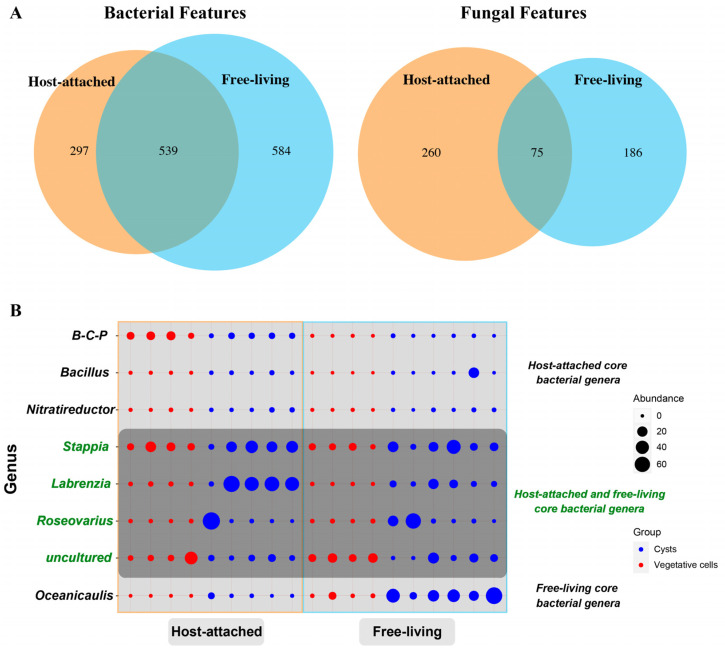
(**A**) Venn diagram showing the numbers of shared and unique bacterial and fungal features. (**B**) Core bacterial genera associated with *Scrippsiella acuminata*. Relative abundance of core bacterial genera found in all samples (the middle part, dark gray) and those found in either host-attached (the upper part, light gray) or free-living (the bottom part, light gray) samples. The size of the solid circle represents abundances of core bacterial genera, and the color represents different life-history states, with blue representing the cysts and red representing the vegetative cells. Note: B-C-P, *Burkholderia-Caballeronia-Paraburkholderia*.

Based on centrifugation, filtering, washing, and incubation with Tween-80, EDTA, and SDS, we separated the bacteria and fungi in the culture into a “host-attached” group and “free-living” group (see the Materials and Methods below for more details). Our results indicated that the host-attached and free-living groups contained most overlapping bacterial and fungal features, although some samples were overwhelmed by the dominant species (Figure 2A). For the features detected in the 16S dataset, 539 were shared between host-attached and free-living groups, while 297 were unique to the host-attached group and 584 to the free-living group. For the fungal features detected in the ITS dataset, 75 were shared by both the host-attached and free-living groups, while 260 were unique to the host-attached group and 186 to the free-living group. According to the definition of core genera, four bacterial genera were identified as core bacterial genera associated with *S. acuminata*, including *Stappia*, *Labrenzia*, *Roseovarius*, and an uncultured (thus unidentified) genus (Figure 2B), as they were present in all the samples with a minimum relative abundance ≥0.1%. The four core genera accounted for 46.4% of the whole bacterial features, in which *Stappia* was the most abundant, comprising 32.31% of the core bacterial features, followed by *Labrenzia* (30.29%) and *Roseovarius*, (19.15%). In addition, three bacterial genera, *Burkholderia-Caballeronia-Paraburkholderia* (a group of genera that are closely related taxonomically, which are all originally part of the *Burkholderia* genus), *Bacillus*, and *Nitratireductor*, presented in all host-attached communities, with the abundance of all higher than 0.1% of all the reads (Figure 2B). One bacterial genus, *Oceanicaulis*, was detected from all the samples of the free-living group with an abundance ≥0.1% (Figure 2B). We also searched for the possible core bacterial taxa present either in all vegetative cell samples, or resting cyst samples, or in both life cycle stages. Seven genera were identified as the “core genera” in vegetative cells, with *Marinovum*, *Altererythrobacter*, and *Ponticoccus* being the most abundant (Figure S2), while no genus could be convincingly identified as the core genus from the resting cysts samples.

Regarding the identification of the core fungal genera, we did not identify any single genus of fungi as a core genus that presented in all life stages and/or cultured conditions. However, the genus, *Malassezia*, was detected to present in all the host-attached samples, with an abundance higher than 0.1%, while the two genera, *Malassezia* and *Alternaria*, were found to present in all samples of vegetative cells, with an abundance ≥0.1%.

### 3.3. Comparison Between the Host-Attached and Free-Living Microbiome Communities

**Figure 3:**
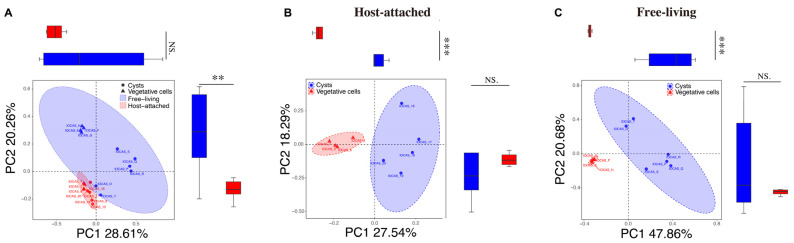
Principal coordinate analysis (PCoA) of bacterial communities based on unweighted unifrac distances. (**A**) All the samples in host-attached and free-living taxa; (**B**) samples in host-attached bacteria (including vegetative cells and cysts); (**C**) samples in free-living taxa (including vegetative cells and cysts). Symbols **, and *** indicate the difference at significant levels with *p* < 0.01, and *p* < 0.001, respectively. NS indicate no significant difference.

While no significant difference was detected in the alpha diversity indices (Shannon-Wiener index, Simpson index, and Observed species richness) between the host-attached and free-living groups (Figure S3), the principal coordinate analysis (PCoA) plot of bacterial features revealed that all the samples formed two distinct clusters according to the degree of associated tightness with microalgal cells, although that for the free-living group was comparatively expanded more loosely (Figure 3A). More importantly, the samples were further clustered into two clusters, one for the vegetative cells and the other for the cysts group (Figure 3B,C). Similarly, a PCoA plot of fungal features also demonstrated two distinct clusters for the host-attached and free-living samples, with two samples as outliers (Figure S6A).

**Figure 4:**
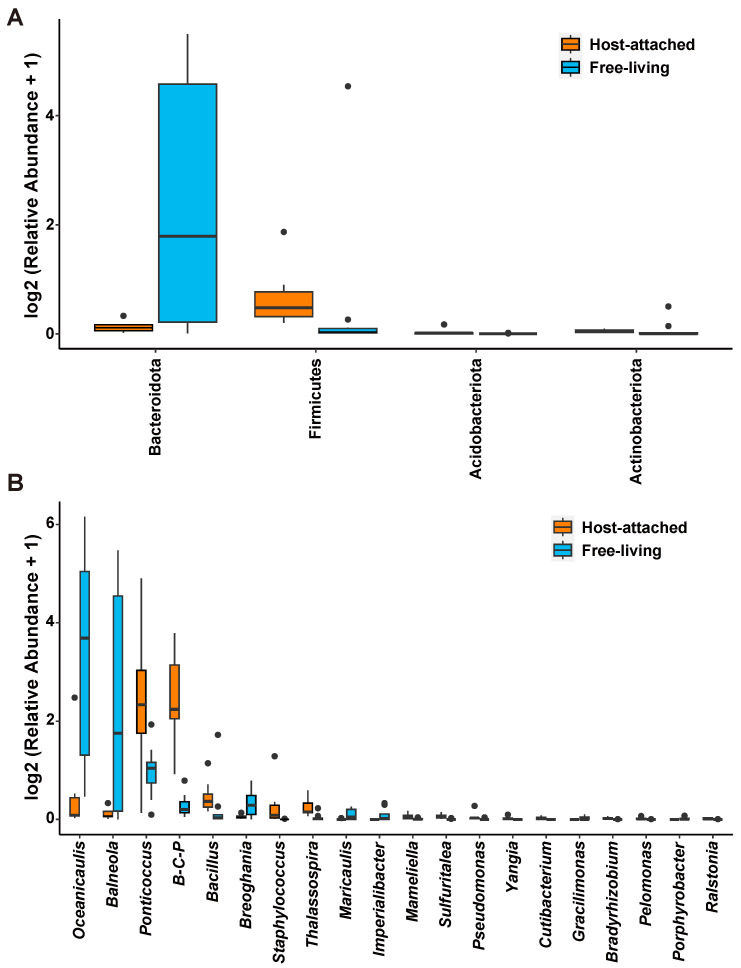
Bar plot of bacterial phyla (**A**) and genera ((**B**), top 20) showing significantly different abundances between host-attached (orange) and free-living (blue) groups. Note: B-C-P, *Burkholderia-Caballeronia-Paraburkholderia*.

A comparison between the host-attached and free-living communities identified the important bacteria and fungi that play vital roles in different groups. At the phylum level of bacteria, the abundance of Bacteroidota, Firmicutes, and Actinobacteriota in the free-living group was significantly higher than that in the host-attached group (Figure 4A), while the abundance of Acidobacteriota was higher in the host-attached group (Figure 4A). At the genus level of bacteria, the host-attached group was enriched in 17 genera, particularly *Ponticoccus*, *Burkholderia-Caballeronia-Paraburkholderia*, *Bacillus*, *Staphylococcus*, and *Thalassospira* (Figure 4B), while the free-living group had a higher abundance of 16 genera, particularly *Oceanicaulis*, *Balneola*, *Breoghania*, and *Maricaulis* (Figure 4B). At the phylum level of fungi, no significant difference in the phyla composition between the host-attached and free-living groups was observed, while, however, the two classes, Tremellomycetes and Ustilaginomycetes, were more abundant in the host-attached group (Figure S7A). At the genus level, the host-attached group had a significantly higher abundance in an unclassified genus of Microascaceae, but lower abundance in *Rhodotorula*, *Bionectria*, *Cephaliophora*, *Ascotricha*, *Cyphellophora*, *Petromyces*, and an unclassified genus of Chaetothyriales (Figure S7B).

**Figure 5:**
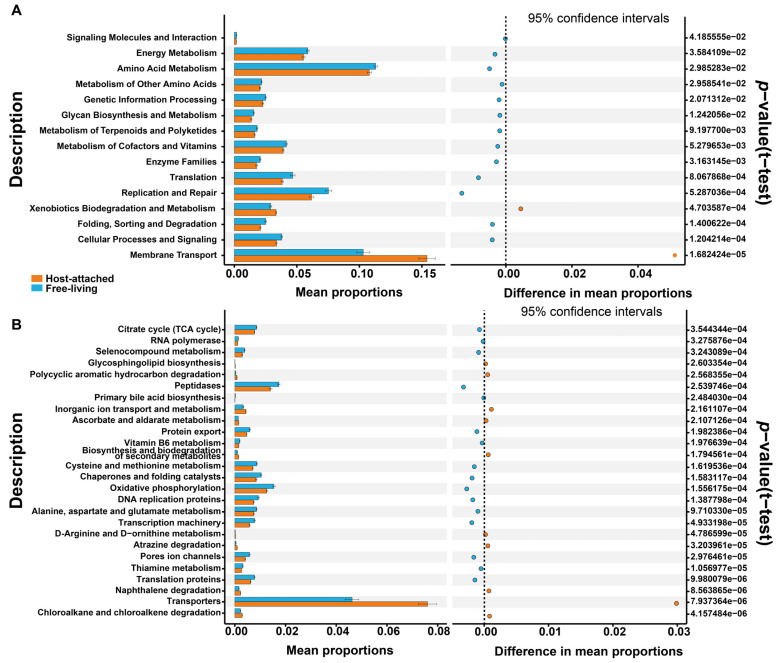
Predictions of the differential functions of bacterial associations between the host-attached (orange) and free-living (blue) groups in KEGG categories at level 2 (**A**) and level 3 (**B**).

The potential functions of bacterial and fungal communities differed between the host-attached and free-living groups, based on PICRUSt2 inference. The KEGG database-based functional prediction of bacterial communities revealed that four functional categories had a higher abundance in the host-attached group at KEGG level 2, whereas 16 categories were more abundant in the free-living group (Figure 5A), including amino acid metabolism, the metabolism of other amino acids, glycan biosynthesis and metabolism, the metabolism of terpenolds and polyketides, metabolism of cofactors and vitamins, and environmental adaption. At KEGG level 3, the host-attached group had a higher abundance in 12 categories of functions and lower abundance in 18 categories of functions (Figure 5B) than that in the free-living group. The functional predictions of fungal communities based on a MetaCyc metabolic pathway database observed that, among the 20 functional categories that were detected with significant differences between the two groups, 14 categories had a higher abundance in the host-attached group, while 6 were higher in the free-living group (Figure S8). These results of functional prediction imply that these two communities differed in their ability to utilize different nutrient forms, with the free-living group having a greater ability to metabolize non-carbon compounds.

### 3.4. Comparisons of the Bacterial and Fungal Communities Among Different Life Cycle Stages

In the PCoA diagram for different samples from the host-attached group and free-living group, the vegetative cells (triangles) and cysts (circles) tended to form distinct clusters (Figure 3A and Figure S6A). A comparison between the vegetative cells group and cysts group for their host-attached and free-living bacterial communities, respectively, revealed that in the host-attached community, the Shannon-Wiener index and the observed species richness of the bacteria–fungi communities in the vegetative cells group were both higher than that in the cysts group (*p* < 0.05, one-way ANOVA), suggesting that the vegetative cells of the host dinoflagellate supported a more active and taxonomically wide-ranging growth of bacteria–fungi communities, while no significant difference in the Simpson index was observed (Figure S4A–C); in the free-living community, the observed species richness in the vegetative cells group was significantly higher than that in the cysts group (*p* < 0.05, one-way ANOVA), but no significant difference was observed in the Shannon-Wiener index and Simpson index between that in the vegetative cells and that in the cysts groups (Figure S4D–F). There was no significant difference in the alpha diversity indices (Shannon-Wiener index, Simpson index, and Observed species richness) between the vegetative cells group and cysts group for their fungal communities (Figure S5). The PCoA plots exhibited that, in both the host-attached and free-living bacterial group, the bacterial compositions in the vegetative cells group were significantly different from those in the cysts group (*p* < 0.05, Figure 3B,C); and the feature fungal compositions also differed between the vegetative cells group and the cysts group (Figure S6B,C).

**Figure 6:**
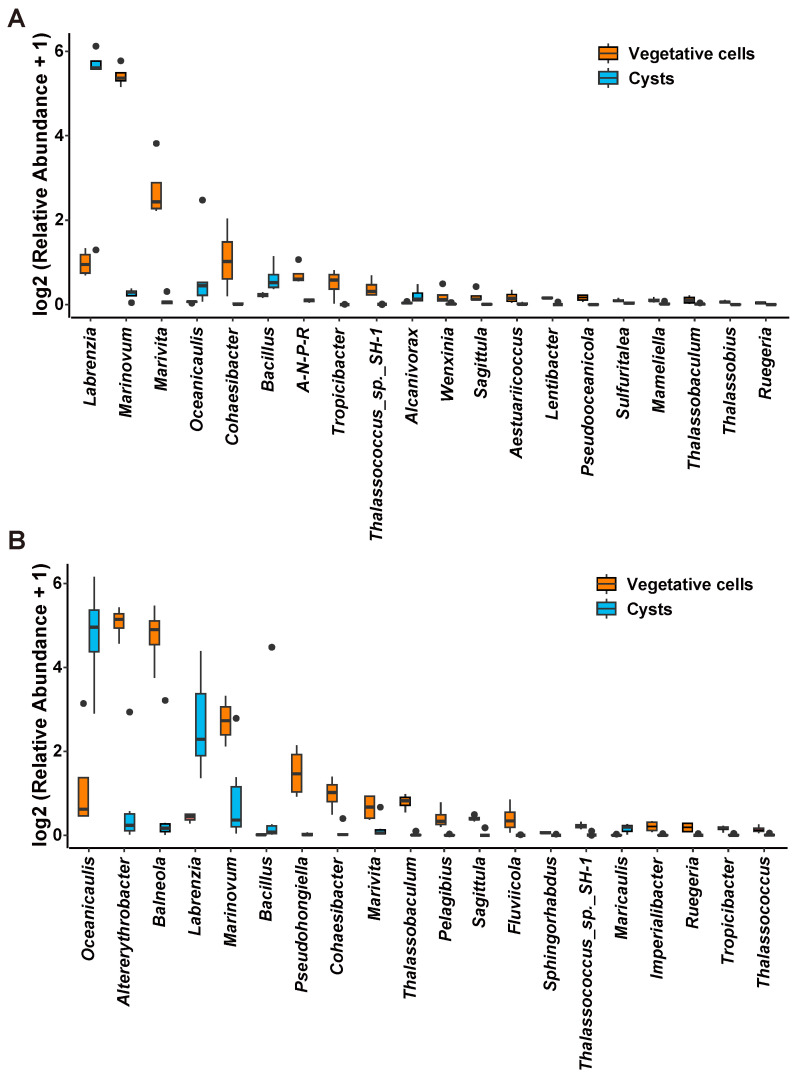
Bar plot of bacterial genera showing significantly different abundance between vegetative cells (orange) and cysts (blue) samples of host-attached (**A**) and free-living groups (**B**). Note: A-N-P-R, *Allorhizobium-Neorhizobium-Pararhizobium-Rhizobium*.

From both the host-attached and free-living microbial samples, we found that three bacterial genera, *Labrenzia*, *Oceanicaulis*, and *Bacillus*, were significantly more abundant in the association with the cysts than that with the vegetative cells (Figure 6). For the host-attached bacteria, the genera *Marinovum*, *Marivita*, *Cohaesibacter*, *Tropicibacter*, *Thalassococcus*, and 22 other genera were more abundant in the vegetative cells group than in the cysts group (Figure 6A), while for the free-living bacteria, the genera *Altererythrobacter*, *Balneola*, *Marinovum*, *Pseudohongiella*, *Cohaesibacter*, *Marivita*, *Thalassococcus*, *Sagittula*, and 35 other genera were more abundant in the vegetative cells group than in the cysts group (Figure 6B). For the fungal communities, the two genera, *Paecilomyces* and *Thermoascus* were significantly more abundant in the cysts group than in the vegetative cells group; in contrast, six genera were more abundant in the host-attached group of the vegetative cells samples than in the cysts samples, and four other genera were significantly more abundant in the free-living group of the vegetative cells samples (Figure S9).

The predicted functions also differed between the bacteria associated with vegetative cells and those with the cysts: According to the categorization in the KEGG database, 10 function categories at KEGG level 2 were more abundant in the vegetative cells group, while 6 categories were more abundant in the cysts group (Figure S10A); further, 21 function categories at KEGG level 3 were more abundant in the bacteria associated with the vegetative cells, while 9 other function categories were more abundant in those associated with the cysts (Figure S10B). For the fungal functions, according to the MetaCyc metabolic pathway database, the vegetative cells group hosted a higher abundance of the sulfate reduction I (assimilatory) than the cysts group (Figure S11).

## 4. Discussion

### 4.1. Core Bacteria–Fungi Genera Stably Co-Existed with S. acuminata

Microbial communities in the aquatic environment are known for their high complexity and diversity, consisting of hundreds to thousands of species [96]. Therefore, the relationship between microalgae and microbiomes are also diverse and often highly complex [2]. Core microbiomes provide a relatively “simple” insight into the interaction between microalgae and its related microbiomes, which is beneficial for the understanding of the highly specific partnerships between a single host and microbes with specific characteristics and a better characterization of the functional importance of microbes [96]. However, our knowledge about whether each microalga hosts characteristic microbiomes and the host–microbiome relationship is changing, but that of the transition of the life cycle stages of the host microalga is still in its infancy, which is why we used the ecologically important dinoflagellate *S. acuminata* as a representative microalgal host to look into this intriguing mutualistic relation.

In this study, we found three core bacterial genera, *Stappia*, *Labrenzia*, and *Roseovarius*, stably coexisted with *S. acuminata* in all the samples (i.e., both in vegetative cells and cysts and both in the host-attached and free-living samples). These core bacterial genera all have been reported to have diverse and flexible metabolic capabilities [97,98,99]. Amongst the three genera, *Stappia* was the most abundant, followed by *Labrenzia* and *Roseovarius*. The *Stappia* was observed to have metabolic versatility, which has been known to be capable of oxidizing carbon monoxide and possess the gene of ribulose 1,5- bisphosphate, suggesting its capability of lithotrophic or mixotrophic metabolism [97,98,99]. Moreover, *Stappia* has a strong survivability in utilizing numerous organics (such as, sugars, organic acids, amino acids, aromatics, and aromatics), surviving at low to high salinities (5–45), being able to respire nitrate or denitrify, and participating in carbon and nitrogen cycling [98]. The second genus, *Labrenzia*, is also a mixotroph and can grow through photosynthetic autotrophy and thus win out over bacteria, being strictly heterotrophic [100,101]. The third, *Roseovarius,* taxonomically classified into the Roseobacter clade, has been widely reported to possess aerobic anoxygenic photosynthesis [102,103] and even metabolize polyhydroxybutyrate and catabolize organic sulfur compounds [104]. The diverse and flexible metabolic capabilities should have contributed to the stable coexistence of these core genera along with the whole life cycle and different physiological conditions of the host dinoflagellate *S. acuminata*.

The consistent coexistence of these three core bacteria with *S. acuminata* suggests that they share a closer association beyond merely temporal and spatial proximity. As found in Kieft et al. [105], different phytoplankton hosts secrete different types of exudates and lysates to attract taxonomically distinct sets of heterotrophic populations or species. Another study has found that *Margalefidinium polykrikoides* and *Alexandrium monilatum* shape different estuarine microbiome communities [15]. Therefore, the available exudates and lysates from *S. acuminata* might have supported the consistently stable coexistence of these three core bacterial genera. On the other hand, many studies have proved that bacteria can help phytoplankton hosts regenerate inorganic nutrients, acquire iron, and provide vitamins for host cells to maintain a more intimate relationship with the microbiomes [1,2,7,8,9,10,11,12]. For example, many Roseobacter clade species including *Roseovarius* have been reported to produce vitamins B_1_ and B_12_ [11], which play important roles in the growth of the host *S. acuminata*. Our results enrich the insights into the closer relationship between host dinoflagellates and their core bacteria genera, which bring important implications for further understanding the association between dinoflagellates and microbiomes.

As described in the results, no single genus of fungi was identified as a core genus that presents in all life stages and/or cultured conditions of *S. acuminata*, while the genus, *Malassezia*, was detected to present in all the host-attached samples, and two other genera, *Malassezia* and *Alternaria*, were found to present in all the samples of vegetative cells. Without speculating too much, we simply, but parsimoniously, assume that this was due to the lacking of a close functional mutualism between the dinoflagellate and its fungal microbiome.

### 4.2. Host-Attached and Free-Living Microbiomes Showed Different Nutritional Preferences

According to their distances to the host cells, we categorized the bacteria–fungi assemblages into two groups: the host-attached and the free-living groups, with the former being physically attached to the host cell, no matter whether they are inside or outside of the host, or symbiotic or opportunistic. Previous analyses for the differences between host-attached and free-living microbial communities have observed that they have different physiological status and activities, with the former always having a closer association with the host cell in functions such as substance exchange [106,107,108]. Nevertheless, studies on dinoflagellate-associated microbiomes have mainly focused on the general term “associated”, rather than to distinguish the host-attached from the free-living communities. The studies in the literature have been conducted with a highly limited number of dinoflagellate species, such as *Cochlodinium polykrikoides* (=*Margalefidinium polykrikoides*), *Prorocentrum minimum*, *P. lima*, *P. hoffmannianum* and *P. rhathymum* [38,109,110]. As an important HAB-forming species, *S. acuminata* has not been surveyed for their host-attached and free-living microbiomes, nor in respect to their interactions. Our work tried to characterize these two groups of bacteria–fungi communities regarding their species composition, species richness, and potential functions and interactions with the host dinoflagellate at different life cycle stages.

The *α*-diversity analysis indicated no significant difference in species diversity (richness) between host-attached and free-living bacteria-fungi communities, whereas PCoA analysis revealed that there were significant differences in the community structure. The host-attached communities were mainly copiotrophic, whereas free-living genera were mainly composed of oligotrophs, and they also have different nutritional preferences. The five genera with a significantly higher abundance in the host-attached bacterial communities, including *Ponticoccus*, *Burkholderia-Caballeronia-Paraburkholderia*, *Bacillus*, *Staphylococcus*, and *Thalassospira*, are all generally considered to be hydrocarbon-degrading bacteria, indicating that the host-attached community has a strong ability in carbon metabolism [111]. These five hydrocarbonoclastic bacteria were capable of growing in media with hydrocarbons as the major or sole carbon source (*Ponticoccus* on polycyclic aromatic hydrocarbons, PAHs [35,112]; *Burkholderia-Caballeronia-Paraburkholderia* on toluene [113]; *Staphylococcus* on hexane [114]; *Bacillus* on toluene [115]; and *Thalassospira* on hydrocarbons as the sole carbon and energy source [116,117]). In the fungal communities, we also found that an unclassified genus of Microascaceae was more abundant in the host-attached group, which, surprisingly, was also found to be reactive to low-molecular-weight carbon sources, thus indicating its carbon catabolism ability [118]. In the free-living communities, the richness levels of four bacterial genera (*Oceanicaulis*, *Balneola*, *Breoghania*, *Maricaulis*) and five fungal genera (an unclassified genus of Chaetothyriales, *Rhodotorula*, *Bionectria*, *Cephaliophora*, and *Ascotricha*) were significantly more abundant than in the host-attached group, and were unexceptionally well adapted to the oligotrophic environment and were proficient at metabolizing a variety of substances. The four bacterial genera mentioned above were all reported to have versatile metabolic abilities and alternative carbon and energy reserves, and were highly competitive under oligotrophic conditions [119,120,121,122,123,124,125,126], and the five fungal genera were all reported to inhabit an oligotrophic environment [127,128,129]. Moreover, the results of functional predictions showed that the host-attached and free-living communities were significantly different in the metabolism of various energy source substances, including amino acid metabolism, the metabolism of other amino acids, glycan biosynthesis and metabolism, the metabolism of terpenoids and polyketides, and metabolism of cofactors and vitamins, which hint that these two communities differed in their ability to utilize different nutrient forms.

The host-attached life form is advantageous over the free-living life form in easily accessing resources and protecting against predators and environmental pressures [57,130]. Therefore, the micro-niche provided by the host cell can facilitate microbial diversification, allowing the host-attached microbial communities to be more diverse than the free-living microbial communities [131]. In our study, the community structures of the two communities, albeit having no difference in alpha diversity, varied and were mainly manifested in different nutritional preferences, which agreed with previous field studies showing that host-attached and free-living bacterial consortia favored different nutrient conditions in a natural environment [132,133]. The ability of photosynthetic hosts to shape microbiomes through excreted metabolites represents a mechanism by which microbes with beneficial effects for the host’s growth are preferably recruited. Our findings show significantly distinct taxonomic and functional compositions of the host-attached and free-living microbiomes in association with our laboratory-raised *S. acuminata*, implying that the dinoflagellate host could influence the two groups of microbiomes in different ways, which might in turn have different functions in impacting the host physiology.

### 4.3. Dinoflagellate Resting Cysts in Dormancy Still Interact with Bacteria and Fungi

While our gradient design incorporated multiple parameters (different life cycle stages, incubation time duration, and temperature), the current analysis specifically focused on life cycle transition as the dominant driver of microbiome composition shift. A series of samples comprising multiple levels of each factor (or treatment) as performed in the present work allowed us to reliably draw the most general conclusions by excluding the stochasticity. The *α*-diversity analysis showed that the ASV diversities of bacteria–fungi communities, both in the host-attached and free-living communities, were significantly higher in those associated with vegetative cells than that associated with resting cysts, suggesting that the phycosphere of dinoflagellate vegetative cells could attract more diverse microbiomes. Owing to their active physiology, the vegetative cells produce more metabolites and thus a more nutrient-rich phycosphere [134,135], which provides more energy sources to support diverse microbial consortia. The PCoA analysis could clearly distinguish the vegetative cells group of bacteria–fungi communities from that of the cysts group, indicating that there was a significant difference in the community structure between the two groups. Comparison analysis revealed that three bacterial genera (*Bacillus*, *Oceanicaulis*, and *Labrenzia*) and two fungal genera (*Paecilomyces* and *Thermoascus*) were significantly more abundant in association with the cysts than that with the vegetative cells. All of these five genera were capable of transforming P from unavailable forms (could not be directly used by phytoplankton or plants) into available forms. Among them, the bacterial genus *Bacillus* and the fungal genus *Paecilomyces* were reported as phosphate-solubilizing bacteria and fungi, which were widely reported to exhibit high solubilization capacity for insoluble inorganic phosphates (not available for plants) [136,137,138,139,140,141]. The genus *Labrenzia* was reported to promote the growth of the dinoflagellate *Prorocentrum donghaiense* through degrading glyphosate into inorganic soluble phosphate (can be directly used by dinoflagellates) [142]. The genus *Oceanicaulis* was proved to have efficient phosphate uptake capacity under inorganic nutrient depleted conditions through high-affinity phosphate transporters [122,143]. In addition, the phytase gene responsible for degrading phytic acid into phosphate was detected in the genome of *Oceanicaulis*, *Bacillus*, and *Thermoascus* [122,144,145]. Phytic acids are an important P source, but few phytoplankton were capable of directly utilizing it for lacking phytase [146]. The organisms with phytase production ability can hydrolyze phytic acid to myo-inositol and inorganic phosphates [147], which can be directly used by phytoplankton.

Phosphorus (P) plays an extremely important role in the whole life cycle and growth stages of dinoflagellates, and indeed all phytoplankton [148,149]. In general, phytoplankton, including dinoflagellates, can directly absorb inorganic phosphate to support algal metabolism and growth, while dissolved organic phosphorus generally requires conversion into inorganic phosphate prior to its metabolic assimilation [149]. It was well confirmed that dinoflagellates (vegetative cells) have the capacity to store internal P, which enables them to maintain their growth rate and metabolic level at a relatively stable state in the early stage of phosphorus limitation [149,150,151]. Interestingly, some case studies have also reported that the resting cysts of dinoflagellates still absorb P from the environment. Lirdwitayaprasit, Okaichi, Montani, Ochi, and Anderson [71] determined the cellular P content of *S. acuminata* (=*S. trochoidea*), and found that the total cellular P content in all cysts’ stages was higher than that in vegetative cells. Rengefors et al. [152] compared the P content of *S. acuminata* cysts between P-enriched and P-depleted medium during a 28-day incubation, finding that the P content in the cysts increased under both conditions, and the content of P in cysts in the P-enriched medium was significantly higher than that in the P-depleted medium. Meanwhile, with the P content in cysts increased, the dissolved inorganic phosphate in the medium decreased. Using energy dispersive X-ray microanalysis (XRMA), Rengefors et al. (1999) compared the P content in field cysts from different years, and found that the P content in cysts preserved for one year was significantly higher than that in newly collected cysts, and the P uptake experiment also found that the P content in cysts in P-enriched medium was significantly higher than that in the medium without P [153]. These studies all pointed to the fact that the dinoflagellate cysts still need to absorb P from the environment. It was proposed that the absorbed P during the dormant stage would be used in subsequent germination [152]. Resting cysts of the dinoflagellate *S. acuminata* in our experiment were produced under N and P co-deficiency [77], and the incubation time in the experiment design was long enough to deplete the inorganic P (can be directly used by dinoflagellates) in the medium. We inferred that microbiomes specializing in P solubilization that were enriched in the cysts group might participate in the P absorption of resting cysts during the dormant stage.

The resting stages of phytoplankton exhibit different degrees of metabolic suppression, enabling them to persist for prolonged periods of survival times [59,154,155]. Although at an inactive stage of their life cycle, highly diverse bacterial and fungal taxa were found co-existing with resting cysts of dinoflagellates. Compared with those of vegetative cells, cysts’ associates displayed significant distinctness in community composition, especially with markedly higher relative abundance of microbiomes specializing in P transformation, thus influencing the P availability for phytoplankton. These phosphate solubilizing microbes were inferred to be involved in improving the availability of P (as phosphate) to facilitate the cysts’ phosphorus absorption, and thus to contribute to the dormancy maintenance of dinoflagellates. In contrast to the conventional notion of a resting stage as a stage of inactive metabolism, the most interesting finding in our study suggests that there are still active interactions between resting cysts and phycosphere microbiomes during dinoflagellate dormancy. Our work highlights the complexity of dinoflagellate–microbiome associations/interactions and opens a window to further investigations on the potential functions exerted by these microbes in the course of the dormancy maintenance of dinoflagellates.

## 5. Conclusions

In this study, we investigated the species diversity and composition of the host-attached and free-living bacteria–fungi communities associated with the laboratory-cultured dinoflagellate *S. acuminata* under different life cycle stages. The bacteria–fungi microbiomes revealed high species diversity, with three bacterial genera identified to be the core taxa stably co-occurring with *S. acuminata* and have diverse and flexible metabolic capabilities. A comparison between host-attached and free-living communities also revealed significant differences between these two groups in that the host-attached communities were mainly copiotrophic, whereas the free-living bacteria–fungi communities were mainly oligotrophic, and the two groups also possessed different nutritional preferences. Furthermore, in contrast to those associated with vegetative cells, the microbes associated with resting cysts displayed a higher relative abundance of the genera specializing in phosphorus solubilization, which may play vital roles in improving the availability of P for cysts and thus in the dormancy maintenance of cysts. Our work highlights the complexity of dinoflagellate–microbiome associations/interactions and opens a window to further investigations on the functions provided by these microbes in the course of the dormancy maintenance of dinoflagellate resting cysts.

## Figures and Tables

**Table 1:** Treatment conditions of each sample.

Sample ID	Sample Name	Association with *Scrippsiella acuminata* Cells	Life Cycle Stage	Temperature	Incubation Time
IOCAS_5	V7T15-A	Attached	Vegetative cells	15 °C	7 d
IOCAS_6	V20T15-A	Attached	Vegetative cells	15 °C	20 d
IOCAS_7	V7T21-A	Attached	Vegetative cells	21 °C	7 d
IOCAS_8	V20T21-A	Attached	Vegetative cells	21 °C	20 d
IOCAS_15	C30T4-A	Attached	Cysts	4 °C	30 d
IOCAS_16	C45T4-A	Attached	Cysts	4 °C	45 d
IOCAS_17	C30T15-A	Attached	Cysts	15 °C	30 d
IOCAS_18	C45T15-A	Attached	Cysts	15 °C	45 d
IOCAS_19	C30T21-A	Attached	Cysts	21 °C	30 d
IOCAS_20	C45T21-A	Attached	Cysts	21 °C	45 d
IOCAS_E	V7T15-FL	Free-living	Vegetative cells	15 °C	7 d
IOCAS_F	V20T15-FL	Free-living	Vegetative cells	15 °C	20 d
IOCAS_G	V7T21-FL	Free-living	Vegetative cells	21 °C	7 d
IOCAS_H	V20T21-FL	Free-living	Vegetative cells	21 °C	20 d
IOCAS_O	C30T4-FL	Free-living	Cysts	4 °C	30 d
IOCAS_P	C45T4-FL	Free-living	Cysts	4 °C	45 d
IOCAS_Q	C30T15-FL	Free-living	Cysts	15 °C	30 d
IOCAS_R	C45T15-FL	Free-living	Cysts	15 °C	45 d
IOCAS_S	C30T21-FL	Free-living	Cysts	21 °C	30 d
IOCAS_T	C45T21-FL	Free-living	Cysts	21 °C	45 d

## Data Availability

The raw sequence data has been deposited into the National Center for Biotechnology Information database (https://www.ncbi.nlm.nih.gov/) (accession No. PRJNA1173930 and PRJNA1173954).

## Supplementary Materials

The following supporting information can be downloaded at: https://www.mdpi.com/article/10.3390/microorganisms13061340/s1, Figure S1: Fungal community relative abundance of different phyla (A) and genera ((B), top 20) in the 20 samples; Figure S2: Core bacterial genera in vegetative cell communities and cyst communities associated with *Scrippsiella acuminata*; Figure S3: The alpha diversity analysis of host-attached (blue) and free-living (orange) bacterial (A–C) and fungal (D–F) groups; Figure S4: The alpha diversity analysis of vegetative cells (blue) and cysts (orange) groups of bacterial community, including host-attached (A–C) and free-living (D–F) taxa; Figure S5: The alpha diversity analysis of vegetative cells (blue) and cysts (orange) groups of fungal community, including host-attached (A–C) and free-living (D–F) taxa; Figure S6: Principal coordinate analysis (PCoA) of fungal community based on unweighted unifrac distances; Figure S7: Barplot of fungal classes (A) and genera (B) showing significantly different abundance between host-attached (orange) and free-living (blue) groups; Figure S8: Prediction of the differential function of fungal associations between the host-attached (orange) and free-living (blue) groups from MetaCyc metabolic pathway database; Figure S9: Barplot of fungal genera showing significantly different abundance between vegetative cells (orange) and cysts (blue) samples of host-attached (A) and free-living groups (B); Figure S10: Prediction of the differential function of bacterial associations between vegetative cells (orange) and cysts (blue) groups in KEGG categories at level 2 (A) and level 3 (B); Figure S11: Prediction of the differential function of fungal associations between the vegetative cells (orange) and cysts (blue) groups from MetaCyc metabolic pathway database; Table S1: The valid data statistics of 16S and ITS rRNA gene amplicons sequencing; Table S2: Alpha diversity indices of each sample; Table S3: Annotation and statistics of different classification levels of each sample.
