# Supplementary material for: Transitions of the Bacteria–Fungi Microbiomes Associated with Different Life Cycle Stages of Dinoflagellate Scrippsiella acuminata
Source: Microorganisms. 2025 Jun 9;13(6):1340. doi: 10.3390/microorganisms13061340 (PMC12195044; doi:10.3390/microorganisms13061340)
Supplement: Supplementary file 1 [file microorganisms-13-01340-s001.zip › Supplementary Figures.pdf]

Supplementary Figures for

Transitions in composition and possible functions of the bacteria-fungi microbiomes associated with different life cycle stages of dinoflagellate Scrippsiella acuminata, particularly resting cysts.

Supplementary Figure 1. Fungal community relative abundance of different phyla (A) and genera ((B), top 20) in the 20 samples.

Supplementary Figure 2. Core bacterial genera in vegetative cells communities and cysts communities associated with *Scrippsiella acuminata*.

Supplementary Figure 3. The alpha diversity analysis of host-attached (blue) and free-living (orange) bacterial (A-C) and fungal (D-F) groups.

Supplementary Figure 4. The alpha diversity analysis of vegetative cells (blue) and cysts (orange) groups of bacterial community, including host-attached (A-C) and free-living (D-F) taxa.

Supplementary Figure 5. The alpha diversity analysis of vegetative cells (blue) and cysts (orange) groups of fungal community, including host-attached (A-C) and free-living (D-F) taxa.

Supplementary Figure 6. Principal coordinate analysis (PCoA) of fungal community based on unweighted-unifrac distances.

Supplementary Figure 7. Barplot of fungal classes (A) and genera (B) showing significantly different abundance between host-attached (orange) and free-living (blue) groups.

Supplementary Figure 8. Prediction of the differential function of fungal associations between the host-attached (orange) and free-living (blue) groups from MetaCyc metabolic pathway database.

Supplementary Figure 9. Barplot of fungal genera showing significantly different abundance between vegetative cells (orange) and cysts (blue) samples of host-attached (A) and free-living groups (B).

Supplementary Figure 10. Prediction of the differential function of bacterial associations between vegetative cells (orange) and cysts (blue) groups in KEGG categories at level 2 (A) and level 3 (B).

Supplementary Figure 11. Prediction of the differential function of fungal associations between the vegetative cells (orange) and cysts (blue) groups from MetaCyc metabolic pathway database.

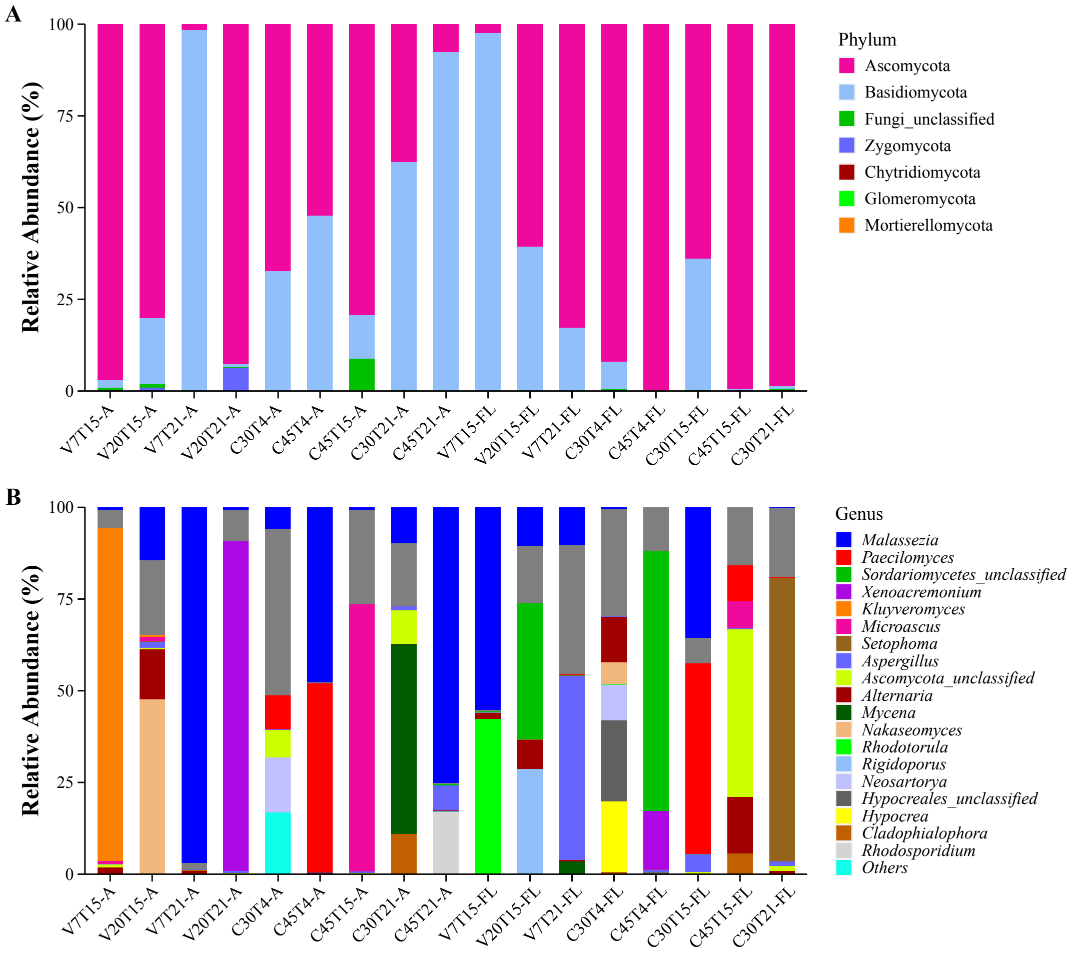

Supplementary Figure 1. Fungal community relative abundance of different phyla (A) and genera ((B), top 20) in the 20 samples. The abundance is presented in terms of percentage in total effective sequences in a sample. The treatment conditions of each sample can be viewed in Table S1, and unsuccessfully amplified samples are not shown.

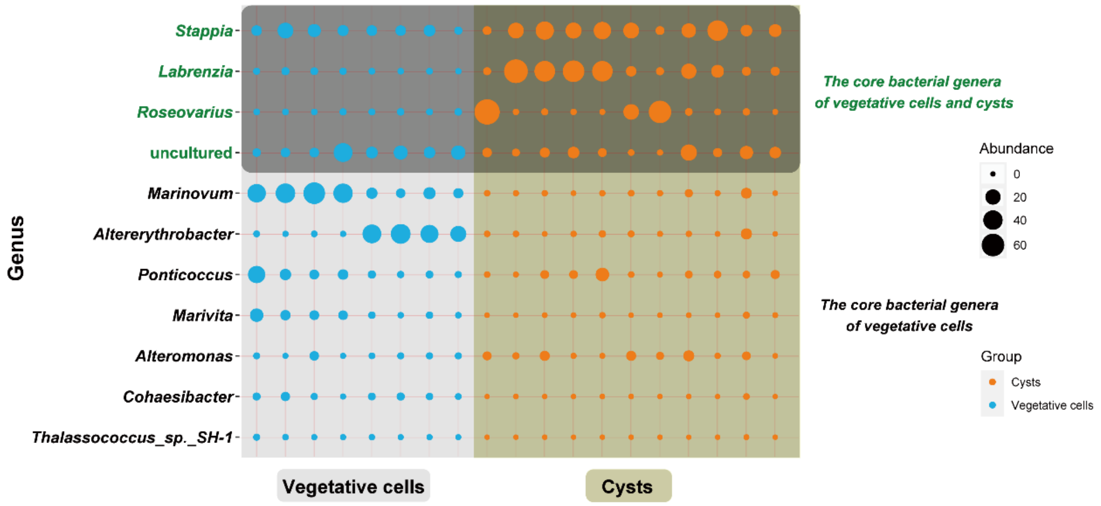

Supplementary Figure 2. Core bacterial genera in vegetative cells communities and cysts communities associated with *Scrippsiella acuminata*. Relative abundance of core bacterial genera found in all samples (the upper part, darkgray) and those found

in vegetative cells (the bottom part, lightgray) samples. The size of the solid circle represents abundances of core bacterial genera, and the color represents different life-history states, with red representing the cysts and green representing the vegetative cells.

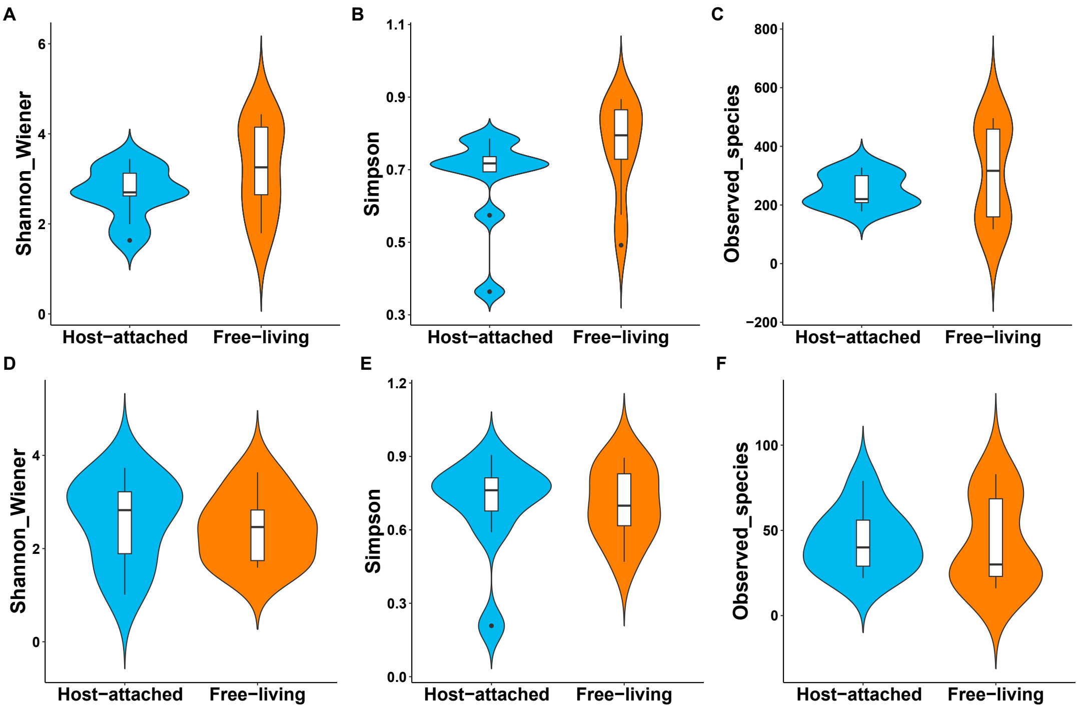

Supplementary Figure 3. The alpha diversity analysis of host-attached (blue) and free-living (orange) bacterial (A-C) and fungal (D-F) groups.

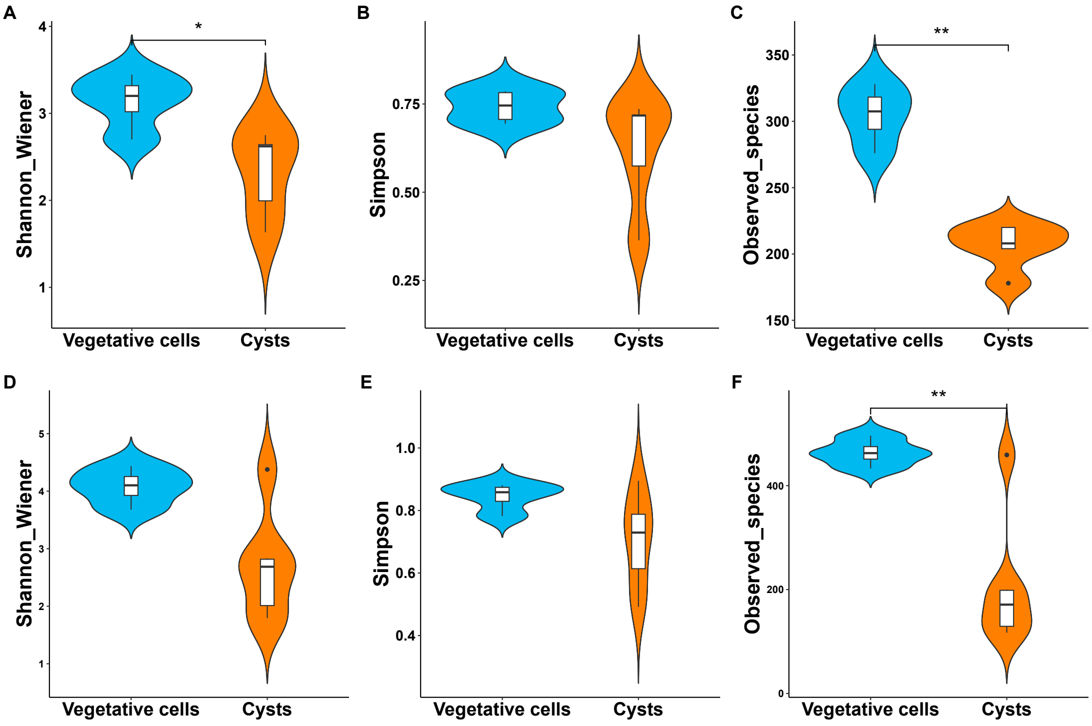

Supplementary Figure 4. The alpha diversity analysis of vegetative cells (blue) and cysts (orange) groups of bacterial community, including host-attached (A-C) and free-living (D-F) taxa.

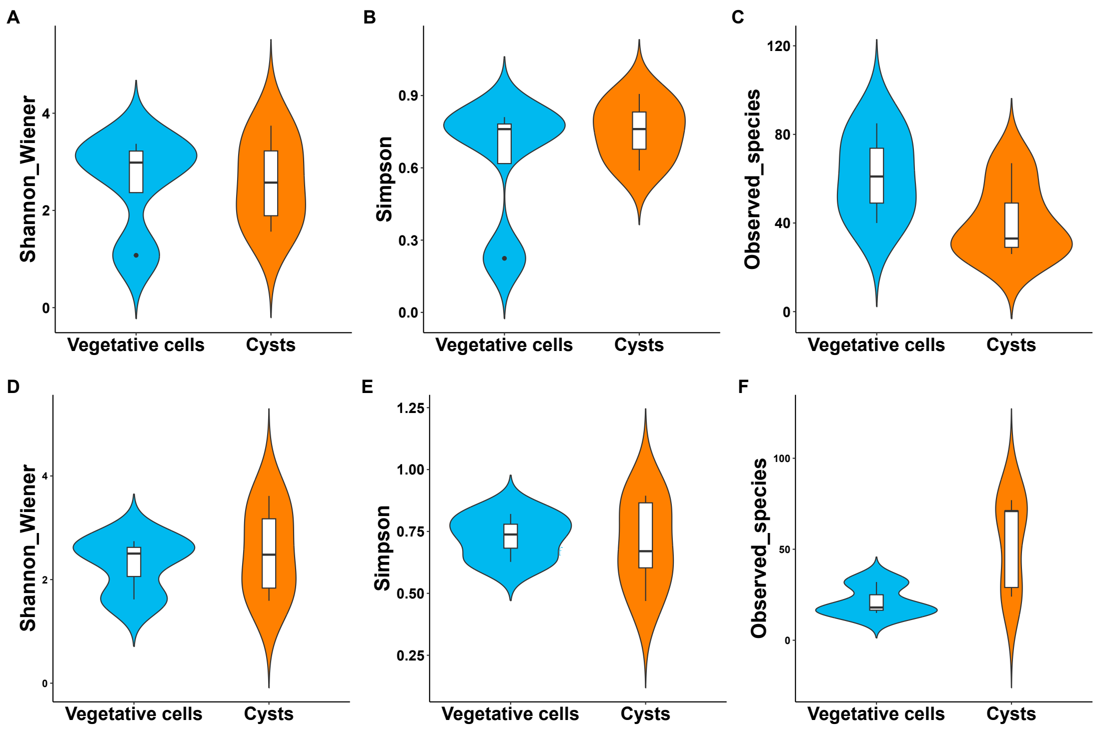

Supplementary Figure 5. The alpha diversity analysis of vegetative cells (blue) and cysts (orange) groups of fungal community, including host-attached (A-C) and free-living (D-F) taxa.

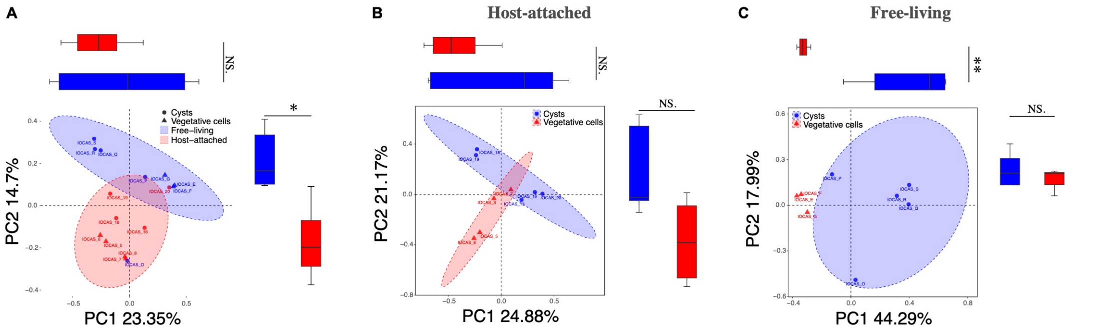

Supplementary Figure 6. Principal coordinate analysis (PCoA) of fungal community based on unweighted-unifrac distances. (A) All Samples in host-attached and free-living taxa; (B) Samples in host-attached taxa (including vegetative cells and cysts); (C) Samples in free-living taxa (including vegetative cells and cysts).

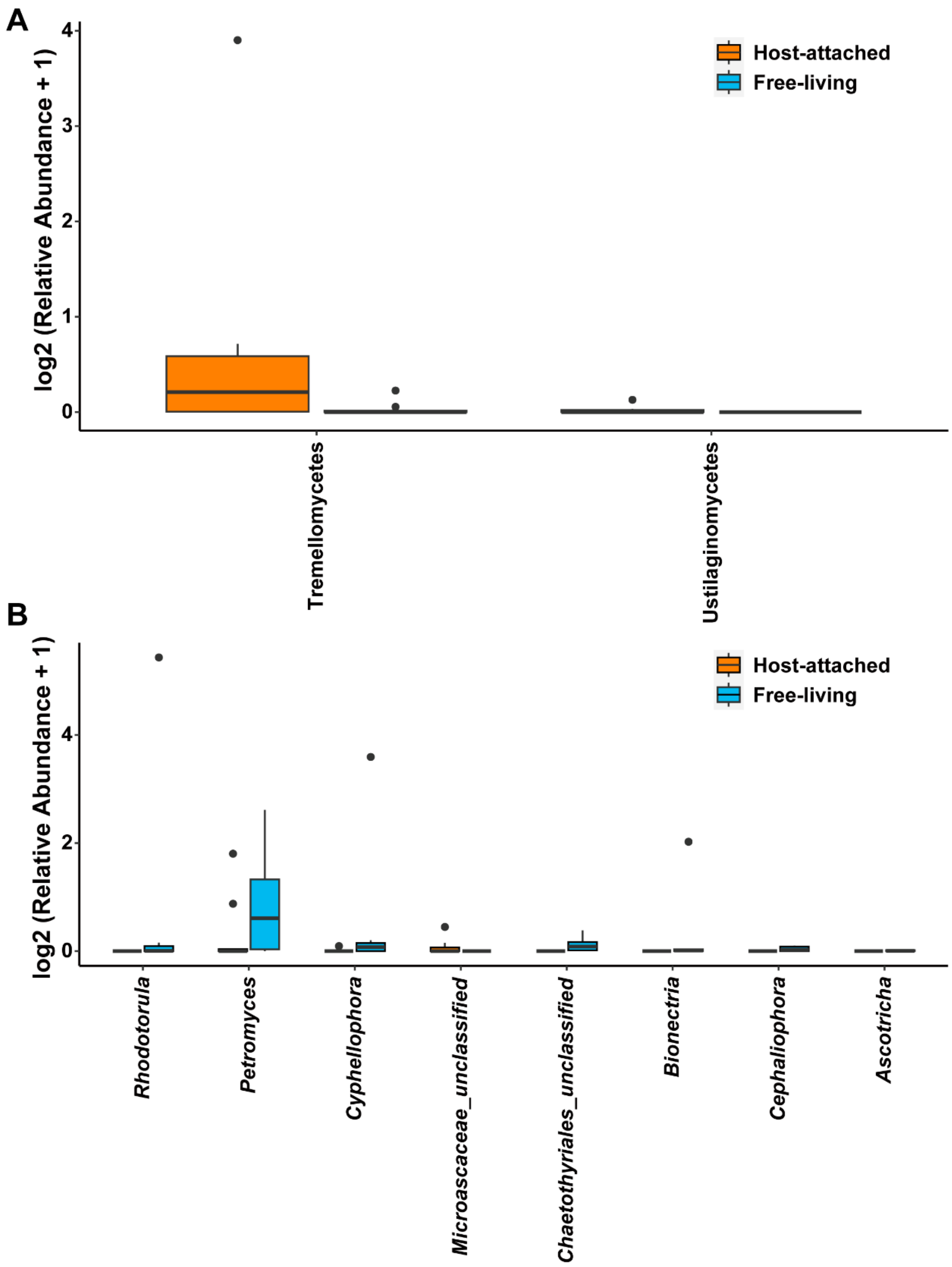

Supplementary Figure 7. Barplot of fungal classes (A) and genera (B) showing significantly different abundance between host-attached (orange) and free-living (blue) groups.

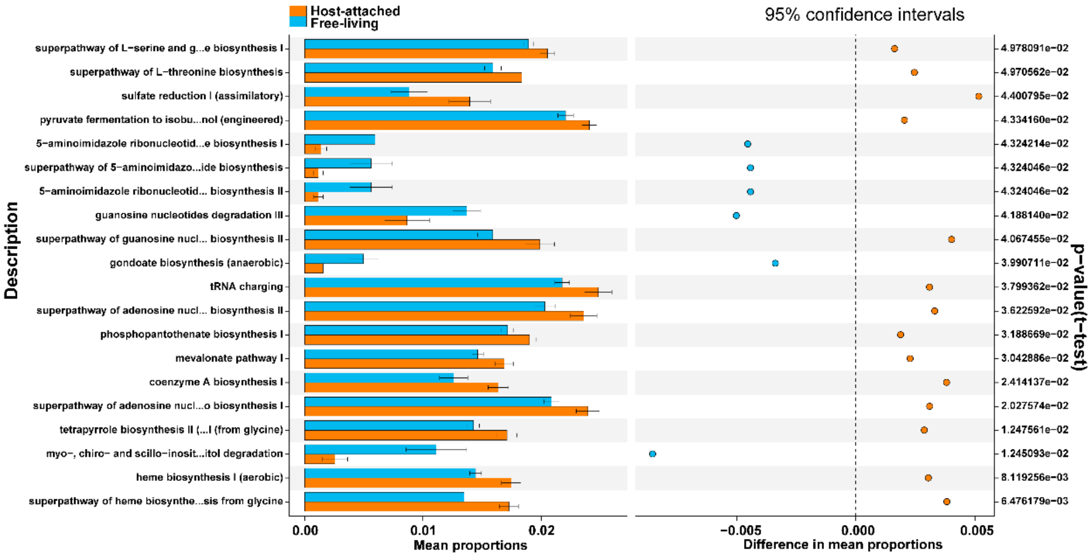

Supplementary Figure 8. Prediction of the differential function of fungal associations between the host-attached (orange) and free-living (blue) groups from MetaCyc metabolic pathway database.

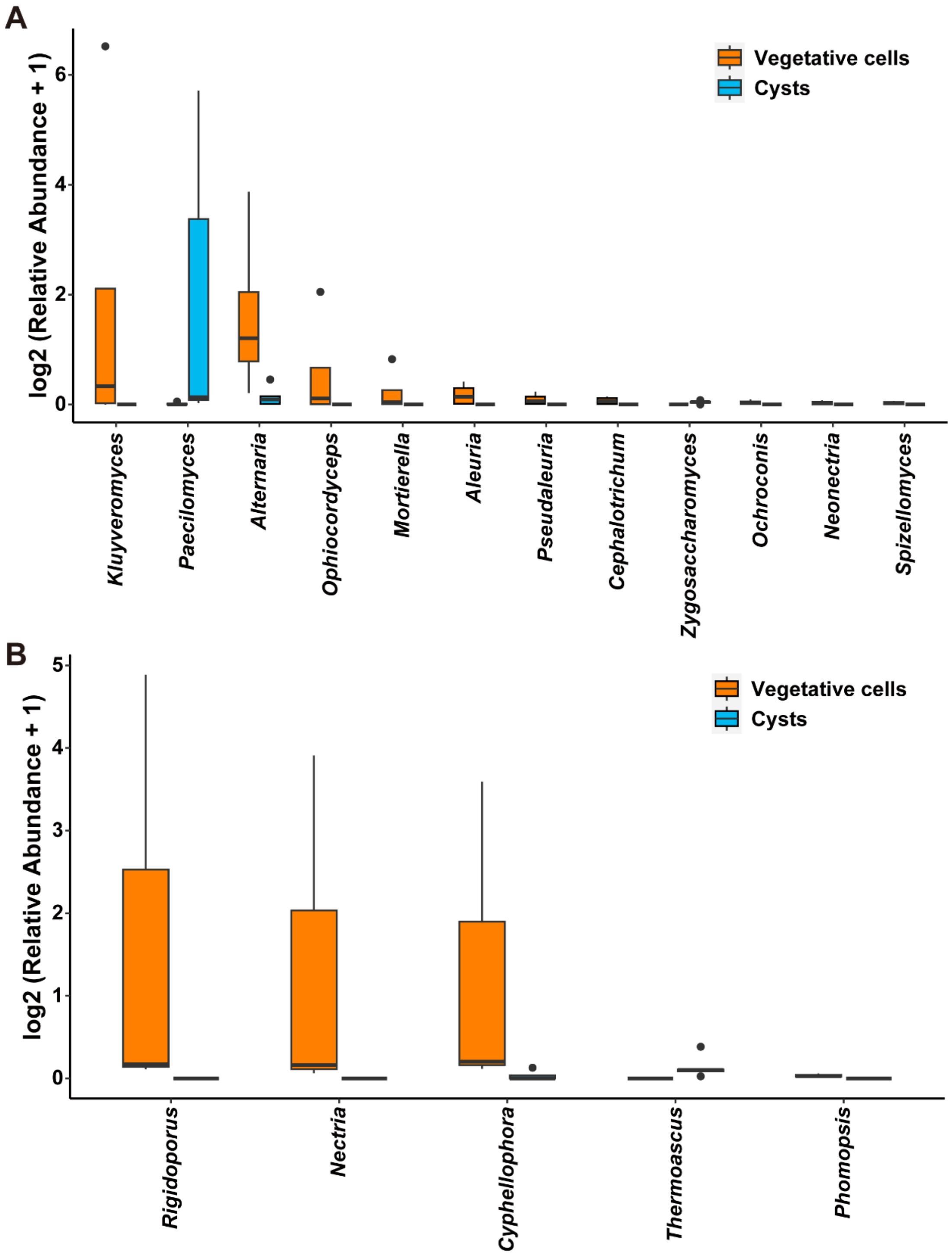

Supplementary Figure 9. Barplot of fungal genera showing significantly different abundance between vegetative cells (orange) and cysts (blue) samples of host-attached (A) and free-living groups (B).

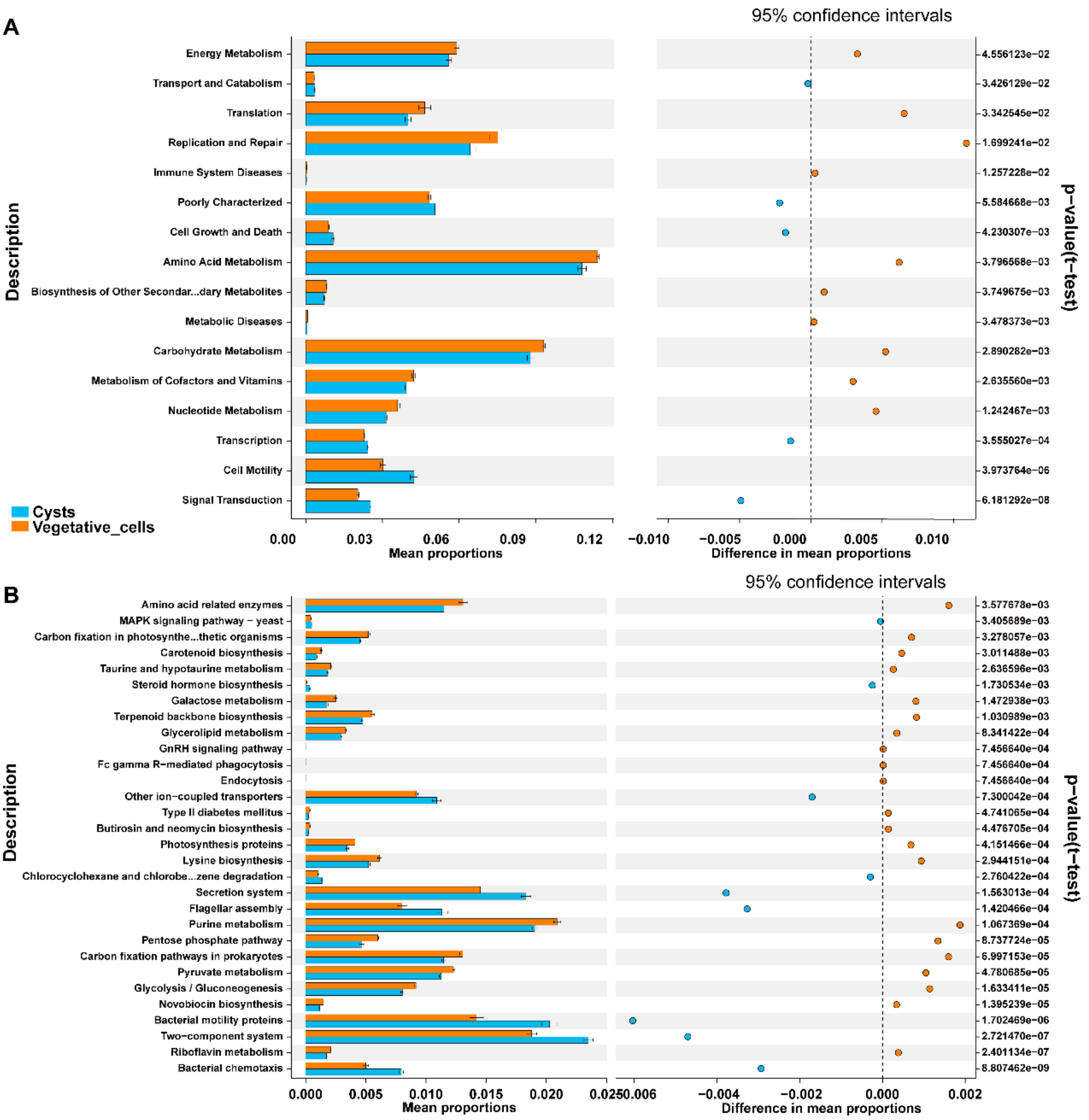

Supplementary Figure 10. Prediction of the differential function of bacterial associations between vegetative cells (orange) and cysts (blue) groups in KEGG categories at level 2 (A) and level 3 (B).

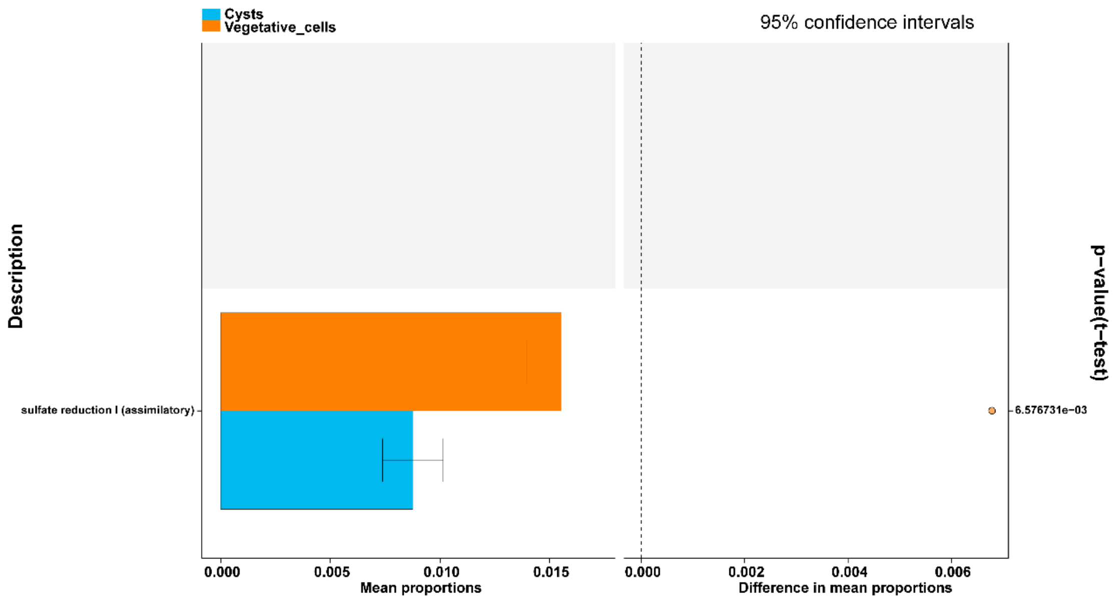

Supplementary Figure 11. Prediction of the differential function of fungal associations between the vegetative cells (orange) and cysts (blue) groups from MetaCyc metabolic pathway database.
